# THE RELATIONSHIPS AMONG VOLUNTEERING, EVERYDAY DISCRIMINATION, AND LIFE SATISFACTION IN TWO AGE GROUPS

**DOI:** 10.1093/geroni/igad104.2845

**Published:** 2023-12-21

**Authors:** Huei-wern Shen, Yi Wang

**Affiliations:** University of North Texas: Denton, Denton, Texas, United States; University of Iowa, Iowa City, Iowa, United States

## Abstract

Experiencing discrimination is known to bring life dissatisfaction across different life stages. While volunteering benefits older adults in many aspects, including life satisfaction, the interplay among discrimination, volunteering, and life satisfaction in different age groups is unknown. To explore such relationships, the present study included a subsample of 5791 respondents who were 51+ from the 2016 Health and Retirement Study; among them, 3191 were 65 years (older group) and older and 2600 were between 51 and 64 years old (younger group). Volunteering referred to doing unpaid work for religious, educational, health-related, or other charitable organizations. Discrimination was measured by the six-item Everyday Discrimination Scale (e.g. In your day-to-day life, how often are you treated with less courtesy or respect than other people) and was dummy-coded due to skewness (never experienced vs. ever experienced). Controlling for health-related variables (e.g., ADLs/IADLs), SES (e.g., income), contextual features (e.g., urban/rural), and sociodemographics (e.g., gender, race), findings from multiple multivariate linear regression models showed that in the overall sample, discrimination was negatively associated with life satisfaction whereas volunteering promotes it. When considering the role volunteering played in the relationships between discrimination and life satisfaction, volunteering in the younger group did not mediate the negative relationship between discrimination and life satisfaction. Volunteering in the older group, however, significantly mitigated the negative impacts of discrimination on life satisfaction. Results highlight the possible extended benefits of volunteering in old age. Future research considering the health impacts of different types and levels of volunteering will be discussed.

